# Conceptualizing the potential of entrepreneurship to shape urban sustainability transformations

**DOI:** 10.1186/s42854-023-00048-w

**Published:** 2023-02-08

**Authors:** Christopher Luederitz, Linda Westman, Alexander Mercado, Aravind Kundurpi, Sarah Lynn Burch

**Affiliations:** 1 Desautels Faculty of Management, McGill University, 1001 Sherbrooke St W, Montreal, Quebec, H3A 1G5 Canada; 2 Urban Institute, University of Sheffield, 219 Portobello, Sheffield, S1 4DP UK; 3 Cornell Cooperative Extension of Suffolk County, 423 Griffing Ave, Riverhead, New York, 11901 USA; 4 SPROUT Lab, Department of Geography and Environmental Management, University of Waterloo, 200 University Ave W, Waterloo, Ontario N2L 3G1 Canada

**Keywords:** Small- and medium-sized businesses (SMEs), Leverage points, Systems transformations, Business sustainability, Internal business transformations, Systems thinking, Place-based entrepreneurship

## Abstract

Entrepreneurship has emerged as a key element for experimentation and niche innovation in sustainability transitions. Yet, its contributions beyond this initial stage and the multi-pronged role that entrepreneurs can play in transformation processes remain elusive. In response, we conceptualize and empirically illustrate how entrepreneurs can contribute to innovations within firms and to city-wide processes of change. With insights from small- and medium-sized enterprises in European and North American cities, we develop a framework encompassing eight intervention types through which entrepreneurs shape urban sustainability transformations. We propose avenues for future research to better understand the distributed role of entrepreneurship and how it can contribute to shaping and accelerating change toward sustainability across integrated levels of urban transformations.

## Science highlights

- Eight intervention types are identified through which entrepreneurs shape urban sustainability transformations
- Interventions span entrepreneurship within firms and at the city-level
- Transformative entrepreneurship moves from isolated interventions to comprehensive system change
- The framework is not a mechanistic toolbox but an invitation to foster critical reflection

## Policy highlights

- Entrepreneurs can support urban transformations beyond the firm level
- They shape material flows, interactions of residents, policy-making, and the identity of entire neighborhoods
- The framework enables practitioners to foster entrepreneurship in support of urban transformations

## Introduction

Entrepreneurship constitutes a pivotal force of urban transformations toward sustainability (Cohen and Muñoz 2015; Gomez et al. 2015). Indeed, urban entrepreneurs involved in economic, social, cultural, and political processes of change, constitute and drive innovations in cities, making these actors uniquely positioned to support urban sustainability transformations (Patzelt and Shepherd 2011; Woolthuis et al. 2013; Muñoz and Cohen 2016). This potential of entrepreneurs to shape urban transformations derives from their intimate knowledge of the local environment, social relationships, and personal aspirations, as well as their embeddedness in material and institutional structures of cities (Murphy 2006; Cohen and Muñoz 2015; Westman et al. 2019).

In the context of urban change, innovations are not only created within firms (Henrekson and Sanandaji 2019) but also require distributed, city-wide processes that support sustainability transformations (Whiteman et al. 2011; Ma et al. 2018; Covin and Wales 2019). While these processes evolve alongside one another and are interdependent, this phenomenon is often separately discussed in the literatures on sustainable entrepreneurship (Schaltegger and Wagner 2011; Muñoz and Cohen 2018) and sustainability transitions (Parrish and Foxon 2006; Hörisch 2015; Bidmon and Knab 2018). The scholarship on sustainable entrepreneurship offers insights into processes of market-oriented value creation, the underpinning personal motivations of entrepreneurs, and their role in organizational change (Schaltegger and Wagner 2011; Muñoz and Cohen 2018). This perspective helps explain the inclination of urban entrepreneurs to pursue goals beyond growth and profits, while such venturing is shaped by personal interactions between stakeholders (Gomez et al. 2015; Muñoz and Cohen 2016; Runyan and Covin 2019). Entrepreneurship, in the context of sustainability transitions, is described as the driving force behind niche innovations, which feed into broader patterns of change in technological and economic systems such as cities (Loorbach and Wijsman 2013; Wolfram et al. 2016; Bidmon and Knab 2018). With its focus on system-wide reconfigurations, this research has illuminated different dimensions of how entrepreneurship can puncture conventional socio-technical arrangements and eventually contribute to large-scale transitions. Yet, as a result of these different approaches to entrepreneurship, the processes underpinning urban transformations are compartmentalized, limiting our understanding of the transformational potential of entrepreneurship and restricting our ability to study evolving change across firm- and city-levels to support innovation for sustainability.

This article integrates these perspectives on entrepreneurship to conceptualize its contributions to urban transformations. We ground this conceptualization in two strands of research, integrating, first, a placed-based inquiry to capture the social embeddedness of entrepreneurship within cities (Shrivastava and Kennelly 2013; Westman et al. 2019; Karvonen et al. 2021). Second, we draw on systems thinking to understand the interconnectivity between entrepreneurial interventions and their urban environments, as well as the complexity of urban transformations toward sustainability (Shrivastava 1995; Abdelkafi and Täuscher 2016; Williams et al. 2019). Building on this rich foundation, we develop a framework to make sense of the transformational potential of entrepreneurship in urban contexts. This framework synthesizes contributions from the scholarship on sustainable entrepreneurship and research on the role of entrepreneurs in societal transformations. We enrich and apply this framework through empirical research on small- and medium-sized enterprises (SMEs) in Vancouver, Toronto (Canada), London (United Kingdom), and Rotterdam (the Netherlands). The contribution of this article is twofold. First, we respond to a key gap in the literature by systematically assessing and defining how entrepreneurial interventions contribute to the sustainability performance of businesses and connecting this impact to city-level change (Muñoz et al. 2018; Lüdeke-Freund 2020). Moreover, by providing empirical illustrations of the framework, we offer practical insights into how a specific actor (i.e., SMEs) supports urban transformations toward sustainability (van der Vleuten 2019; Hölscher and Frantzeskaki 2021). Second, we contribute a new understanding of how entrepreneurship is involved in societal transitions by conceptualizing the distributed, city-wide change processes through which this occurs. Synthesizing these insights, we develop a foundation for future research to study *pathways of change* that describe how entrepreneurship is connected to and co-evolves with processes of urban sustainability transformations. We offer new avenues to explore distributed and co-evolutionary dynamics between firm- and city-level entrepreneurship and the social impact of businesses beyond formal markets.

In the next section, we review the conceptual considerations upon which our framework rests and propose eight categories for studying how entrepreneurial interventions support urban sustainability transformations. After describing the methods used to develop the conceptual framework, we present and enrich our proposal through empirical data based on research in four cities. In Discussion section, we discuss areas of application of the framework to better understand how entrepreneurship contributes to urban sustainability transformations. We conclude with reflections on the significance of this work.

## The multi-pronged role of entrepreneurship in urban transformations

Analyses of entrepreneurship in urban transformations have emerged in two distinct fields of research: the literature on sustainable entrepreneurship and sustainability transitions.

### Perspectives on sustainable entrepreneurship

In research on sustainable entrepreneurship, innovations supporting urban sustainability rest on a notion of entrepreneurship as a “place-based locus of ownership and control, embeddedness or rootedness in the physical, social, and human capital of a place, possessing a sense of place and a social mission” (Shrivastava and Kennelly 2013, p. 90). Cohen and Muñoz (2015, p. 265) suggest that this “new breed of entrepreneur” seeks to not only improve the economic viability of their business but, because of the relationships to the place and people where the business operates, they also strive to generate social and environmental well-being for that community (Thomas et al. 2011; Gomez et al. 2015). The focus of such entrepreneurial venturing is firmly oriented towards addressing urban challenges and improving the quality of life for its citizens (Cohen and Muñoz 2015). This often requires collaboration across businesses, civil organizations, and government units to address challenges such as food security, housing, mobility, gentrification, local job markets, and capacity building (Muñoz and Cohen 2016). Thus, the focus of this literature is on internal processes within firms and their embeddedness in a specific context. There is little effort to systematically comprehend the relationship between individual entrepreneurial ventures and broad directions of urban development. While a growing body of literature is interested in the capacity of sustainable entrepreneurship to shape mass markets (Hockerts and Wüstenhagen 2012; Schaltegger et al. 2016; Westman et al. 2022), this scholarship remains disconnected from analyses of material and institutional landscapes, such as those constituted by urban systems.

### Entrepreneurship in sustainability transitions

The sustainability transitions literature, on the other hand, adopts a systems-based view of societal change, in which entrepreneurs play a leading role in introducing novelty. Broadly speaking, this scholarship frames entrepreneurship as a source of innovation, frequently with a narrow focus on technology. This perspective has helped to explain how entrepreneurship emerges and matures in protected spaces (niches) and eventually contributes to establishing alternative socio-technical configurations (regimes) (Rip and Kemp 1998). Entrepreneurs are seen as conductors of experiments (in niches), through which they develop technology-focused innovations, introduce novelty, and deviate from conventional ways of doing (Smith 2007; Geels 2011). The assumption is that “small-scale experiments create diversity at the niche-level … and scaling up experiments enhances the emergence of a breakthrough” (Rotmans and Loorbach 2010, p. 121; Farla et al. 2012). This perspective is based on theories of co-evolution, which assume that shifts in society are produced through alignments between niches and their broader socio-technical context, including changes in industrial sectors, cultural practices, infrastructures, regulations, and social networks (Dosi and Nelson 1994; Kanger and Schot 2019). In the context of urban transformations, co-evolution suggests reciprocal relationships between entrepreneurs, innovations, and cities, and changes in one area will shape dynamics in another. In conclusion, the transitions literature portrays entrepreneurship as activities that are small in scale (Rotmans and Loorbach 2010), happen at the niche level (Smith 2007; Geels 2011), and bring about technological innovations through experimentation. Thus, the emphasis is on the ability of entrepreneurs to generate diversity within a system, rather than their capacity to contribute to the embedding of innovations in institutional or material environments. While increasingly attention has shifted toward how individual actors shape transformations (Schot et al. 2016; van der Vleuten 2019), the particularities through which specific systems, such as cities, shape the pathways through which entrepreneurs enact co-evolutionary change remain untraced.

### A systems perspective for understanding entrepreneurial interventions

Research on urban entrepreneurship often adopts a complex systems perspective – explicitly or implicitly – to examine the interactions and interconnectedness of these actors in and with society (George and Bock 2011; Starik and Kanashiro 2013; Lüdeke-Freund 2020). As such, it relies on approaches related to ‘systems thinking’ that describe patterns of interaction and emerging properties of systems (such as an organism, an ecosystem, a business, a city, or an economy) (Forrester 1971; Checkland 2000). Against this background, we offer our attempt to develop a more fulsome understanding of entrepreneurial actions in urban transformations toward sustainability.

Understanding sustainable entrepreneurship from a systems perspective facilitates strategic consideration of the type of interventions that firms can pursue to foster systemic change. The idea of identifying places to intervene in systems was pioneered by Donella Meadows’ (1999) work on leverage points, and her observations that small interventions in one area can lead to transformations in multiple system elements, properties, and processes. While Meadows initially described the nature of twelve leverage points without specifying the level of application, others aggregated her work into four generic system characteristics (Abson et al. 2017) as target areas for interventions (Luederitz et al. 2017). In this context, interventions refer to actions that can create change in elements, properties, or processes of a system. Accordingly, interventions can be categorized based on their ability to induce change in system characteristics, including:

- Resource interventions: Adjustments that change “mechanical” or quantifiable characteristics, physical structures, and efficiency of processes such as reducing waste or modifying energy and material flows,
- Transactive interventions: Alterations that change the “interactions between elements of a system […] allowing existing processes and structures to adapt more quickly” and become more effective by, for example, building capacities in employees or developing cross-sectoral partnerships,
- Organizational interventions: Reconfigurations that change the design “of the system, how and by whom the system is managed and organized” and the level of agency people have over outcomes, such as developing new or changing existing formal and informal norms as well as governance processes, and
- Value interventions: Transformations that change the intent of the system, shifting its “underpinning beliefs, mindsets and goals” such as changing the ends to why business activities are pursued or remodeling the identity of organizations or collectives that informs their actions (Luederitz et al. 2017, p. 395).

**Fig.1:**
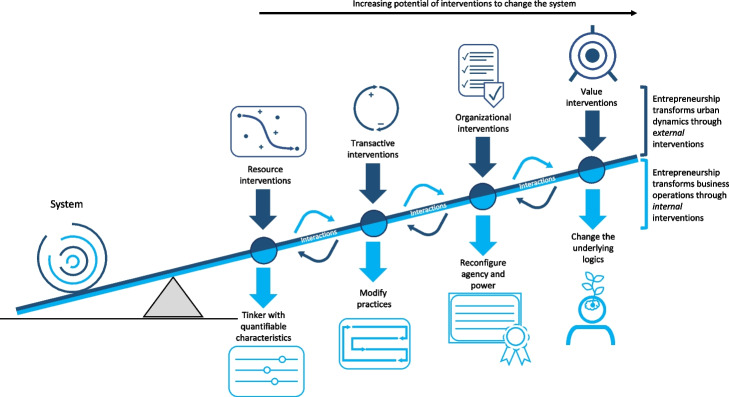
A visualization of how entrepreneurship shapes urban sustainability transformations. The framework depicts two levels at which sustainable entrepreneurship intervenes: the *business level* (light blue) and the *city level* (dark blue). Across these two levels, we identified four interventions (*resource, transactive, organizational, and value interventions*) through which sustainable entrepreneurs change business operations and urban dynamics. The higher an intervention is positioned on the seesaw, the greater its potential to initiate ripple effects throughout a given system aside from changing the properties that are targeted. The framework offers a heuristic tool for researchers exploring the bidirectional *interactions* between firms and the urban context in which they operate to both rigorously examine and explicitly support change for urban sustainability

Structuring and understanding the multi-pronged role of entrepreneurship in changing system characteristics requires contextualizing interventions in relation to the bidirectional line of influence between firms and the urban context in which they operate (see Fig. 1). Next, we explain the research methods to accomplish this contextualization, and, in Structuring the transformational potential of entrepreneurship for urban sustainability transformations section, we define each intervention in-depth in relation to the context of the firm level and the urban context.

## Materials and methods

The methodological approach of this study is based on the principles of analytic induction (Robinson 1951). Analytic induction departs from a rough definition and preliminary conceptualization of a phenomenon, followed by examinations of empirical case(s) to consider how well the explanation captures the phenomenon under study, followed by reconsideration if empirical observation does not match theoretical assumptions (Robinson 1951). For the purpose of building a theoretical framework that captures the connections between entrepreneurship and urban transformations, we began with a systems perspective (Meadows 1999; Abson et al. 2017; Luederitz et al. 2017) and combined it with insights from previous research. These ideas were gradually refined and integrated through our data collection process. Thus, the methodological approach enabled an iterative procedure between gathering empirical data and the process of conceptualization (Bansal and Roth 2000; Hammersley 2011).

### Data collection method

Empirical data on SMEs involved in sustainability-oriented change was collected through in-depth interviews with 150 firms, governments, and non-governmental organizations in four cities, including Vancouver (Canada), Toronto (Canada), London (United Kingdom), and Rotterdam (the Netherlands). We selected SMEs as a focal point of our examination considering they represent the majority of businesses in Canada, provide over 88% of private-sector employment (ISED 2020), emit annually more than 200 million tons of carbon –which is equal to the emissions of Canada’s transportation sector (Climate Smart 2018) – and constitute a private actor that is largely ignored in research on sustainability transitions and entrepreneurship (Burch et al. 2016; Runyan and Covin 2019). The selection of the four urban contexts was informed by a purposeful sampling logic aimed at identifying study sites that could offer deep insight into the subject matter (Patton 2015). Previous research has reported on significant opportunities to learn about SMEs sustainability in Vancouver (Burch et al. 2013), Toronto (Granek and Hassanali 2006; Gomez et al. 2015), London (Revell and Blackburn 2007), and Rotterdam (Whiteman et al. 2011; Loorbach and Wijsman 2013).

A series of semi-structured key informant interviews were carried out in each of the cities (53 in Toronto, 37 in Vancouver, 35 in London, and 25 in Rotterdam). The interviews followed a basic script, which contained questions pertaining to 1) the drivers of sustainability innovation, 2) the patterns of interaction between government and non-governmental actors, 3) the role of SMEs in responding to sustainability challenges, and 4) sources of institutional, cultural, or technical inertia and change. Three categories of participants were invited for interviews, including those who: 1) work for municipal government and who are directly involved in the framing, development, and implementation of sustainability policy; 2) are employed by (or manage) SMEs that have taken a leadership position on sustainability; 3) play a key role in either higher levels of government (i.e., regional/provincial) or in the non-profit sector and have collaborated with municipal staff to develop/implement sustainability policy. Interviews were recorded, transcribed, and coded using NVivo qualitative analysis software based on the iteratively refined set of codes reflecting the intervention types described above.

The selection of case studies in large cities in high-income countries may limit the transferability of findings to areas outside this research context. Entrepreneurship in rural areas, for example, involves different dynamics than those observed in urban environments (Fortunato 2014). Moreover, cultural specificities and social arrangements related to sustainable entrepreneurship differ between places and require researchers to contextualize the framework we develop below (Spence et al. 2011). We have attempted to address some of these issues by complementing the observed dynamics across the four cases with insights from the literature. This supports the contextual application of the framework and situates the cases in our study in relation to broader debates. Future research is therefore tasked with testing and refining the conceptual underpinnings of the framework to develop a better understanding of the implications sustainable entrepreneurship holds for sustainability transformations.

### Framework development

In our case, the purpose of using analytic induction was to build a conceptual clarification of processes of transformational change led by entrepreneurship in urban areas. Our processes for framework development and data collection were therefore conducted in parallel, according to the steps described below.

The first step consisted of identifying the four intervention points described in The multi-pronged role of entrepreneurship in urban transformations section as our point of departure for understanding processes of change unfolding both within businesses and throughout the urban system in which they operate. Next, we searched for relevant businesses that would allow for empirical examination of these assumptions in four cities, including, Vancouver, Toronto (Canada), London (United Kingdom), and Rotterdam (the Netherlands). Relevant firms were identified through an online search of websites (e.g., company websites, sustainability awards, certification schemes) in combination with snowball sampling (following leads from interviewees that suggested other relevant businesses), with the aim of including enterprises that displayed activities relevant to the four intervention points.

The second step (conducted at multiple points throughout the data collection) consisted of reviewing initial cases to contextualize entrepreneurial actions across the four cities. This allowed us to expose gaps in the preliminary framework as some categories were not detected in the empirical data and some entrepreneurial actions were not sufficiently categorized. These insights led to reconceptualizing the framework by reviewing and incorporating relevant literature. For example, we explicitly conceptualized internal business activities to capture resource use, operation, management, and value generation. We also deepened the understanding of how external business activities influence external city dynamics by changing the urban metabolism, governance arrangements, and neighborhood characteristics. At this stage, we refined the definitions of the four initial intervention points based on insights from the entrepreneurship literature (George and Bock 2011). To better reflect the forms of change delivered by entrepreneurship in our research, we reconceptualized the categories as interventions targeting: resource structure, transactive structure, organizational structure, and value structure (see Table 6). The terminology surrounding ‘resources’ was more closely aligned with what we had originally defined as ‘parameter’ interventions (as these relate primarily to physical structures and material resources), while we identified a range of interventions that relate to ‘transactions’ (shifts in patterns of interaction, practices, relations) rather than what was originally defined as feedback loops (see Table 1).

The third iteration consisted of a systematic analysis of our empirical data through qualitative content analysis (Elo and Kyngäs 2008). This was realized by coding the interview material according to the four intervention points, followed by re-coding to complement, adjust, and finalize the categories of intervention. Moreover, for each code we identified an illustrative example that could exemplify each intervention. While these eight cases are presented in Structuring the transformational potential of entrepreneurship for urban sustainability transformations section below, insights from the other interviews are represented in the refined categories of intervention (especially Table 6), as well as in our insights regarding pathways of change.

## Structuring the transformational potential of entrepreneurship for urban sustainability transformations

This section presents the framework for conceptualizing the transformational potential of entrepreneurship for sustainability in cities. We present and discuss the empirical findings to contextualize the four entrepreneurial interventions and relate each intervention to the firm level (internal interventions) and the city-level (external interventions) (see Table 1 and Fig. 1). We enrich the conceptual elaborations with practical examples from our empirical research investigating how firms support sustainability transformations in the cities of Vancouver, Toronto (both Canada), London (UK), and Rotterdam (NL).

**Table 1:** Overview of leverage points, system characteristics and business interventions to mobilize change for sustainability transformations

The 12 leverage points developed by Meadows (1999)	Description of four system characteristics as aggregated by Abson et al. (2017) as target areas for interventions		Business interventions that change internal operation and influence external urban dynamics		Illustrative examples (see Tables 2, 3, 4 and 5)
Constants, parameters, numbers (such as subsidies, taxes, standards)	Parameters	Parameter interventions target the quantifiable properties within a system, such as energy and waste streams or the number of employees. This intervention targets system characteristics described by Abson et al. (2017, 32) as “modifiable” and “mechanistic,” such as a company’s debt, its profit margins, the assets write-off rate or physical elements of a system, such as sizes of stocks or rates of material flows” building on the leverage points of parameters, buffers, and stock-and-flows (see Meadows 1999, 5–8). Generally speaking, related interventions “turn the faucet of environmental degradation” (Meadows 1999: 5), thereby altering the rate or speed of a particular system dynamics without interfering with its nature. This can include tinkering with levels of material consumption (such as increasing efficiency) or the adjustment of social parameters (such as income or paycheck raises). Importantly, parameter interventions do not interfere with behaviors of a system (such as a firm or a city). As a result, these interventions are likely to gain most system impact when they trigger additional interventions that affect the nature of system dynamics.	Resource interventions	Changes that tinker with quantifiable characteristics and increase the efficiency of business operation by doing more with less and reducing generated waste. Related initiatives included clean manufacturing, environmental management, as well as product design and longevity. Businesses may influence external dynamics by reusing and upcycling materials as well as substituting finite for renewable resources as illustrated through closed-loop business models and sustainable urban metabolism.	**Internal operations** • Reduce energy and water usage. • Integrate recycling systems and renewable resources. **External dynamics** • Install pollution prevention systems • Reduce city-wide waste. • Improve quality of natural resources (e.g., natural habitats, water bodies, urban canopy cover).
The sizes of buffers and other stabilizing stocks, relative to their flows.					
The structure of material stocks and flows (such as transport networks, population age structures)					
The lengths of delays, relative to the rate of system change	Feedbacks	Feedback interventions target the relationships between elements that contribute to the nature of system dynamics, such as the practices, routines and knowledge of people. These interventions target system characteristics described by Abson et al. (2017, 32) as “the interactions between elements within a system of interest that drive internal dynamics (e.g., dampening or reinforcing feedback loops) or provide information regarding desired outcomes (e.g., the effectiveness of a given incentive scheme)” building on the leverage points of delays, balancing feedbacks, and reinforcing feedbacks (see Meadows 1999, 8–12). Generally speaking, feedback interventions address the patterns that emerge from a specific set-up but do not interfere with the rules that govern the behavior of a system. Accordingly, related interventions offer people new ways of doing things and support reflections on activities for determining if satisfactory outcomes are generated.	Transactive interventions	Changes that modify practices and interactions of people connected with the business and its product/services. Related initiatives may require education of customers and training of employees in order for a business model to become effective in achieving its goals. Businesses may influence external dynamics by supporting the uptake of sustainability-oriented routines, supporting cross-sectoral partnerships, and delivering product/services that change the cityscape.	**Internal operations** • Develop customer reward systems to alter consumption practices (e.g., bring own mug or reusable bags). • Promote employee behavior change (e.g., carpool; active transport; teleworking). **External dynamics** • Facilitate spaces for young entrepreneurs to develop their businesses and ideas. • Alter practices for developing infrastructure (e.g., sustainable building design).
The strength of negative feedback loops, relative to the impacts they are trying to correct against					
The gain around driving positive feedback loops					
The structure of information flows (who does and does not have access to what kinds of information)	Design	Design interventions redefine structures of agency and authority by reallocating patterns of recognition, resources, and power that determine who controls the governance of the system. This intervention targets system characteristics described by Abson et al. (2017, 32) as being “related to the structure of information flows, rules, power and self-organization,” building on the leverage points of information flow, rules, and self-organization (see Meadows 1999, 12–16). This means that design interventions can change lines of accountability as well as redefine who gets to decide on the appropriateness of actions and what goals are legitimate to pursue. While design interventions can fundamentally alter the organization of a firm or a city, they do not question or change the goal orientation of a system.	Organizational interventions	Changes that reconfigure agency and power through informal and formal rules that realize shared ownership and collaborative decision-making within the business. Firms may influence external dynamics by shaping urban governance and the involvement of firms in establishing rules and building systems of authority in a city.	**Internal operations** • Lead collaborative design with clients to show the environmental impacts of their projects. • Establish new positions to guide sustainability-oriented mandate (e.g., sustainability coordinator). **External dynamics** • Empower marginalized communities and individuals to obtain employment. • Engage with policymakers to influence changes to regulations to improve access to livelihood in a community.
The rules of the system (such as incentives, punishments, constraints)					
The power to add, change, evolve, or self-organize system structure					
The goals of the system	Intent	Intent interventions target the goals of a system, its identity and the values that inform how and what actions are pursued with what intensity. These interventions target system characteristics described by Abson et al. (2017, 32) as “the norms, values and goals embodied within the system of interest and the underpinning paradigms out of which they arise,” building on the leverage points of goals, paradigm, and transcend paradigms (see Meadows 1999, 16–19). Generally speaking, intent interventions target the deepest underlying goals that organize a business as it influences the worldviews and beliefs that shape its operations (e.g., monetary valuation captures something real, growing is good, a business is constituted by people that produce something with everything else being considered as outside) (Meadows 1999). Such interventions may change the purpose of a business (i.e., why a company does business) and the values that inform how a city functions and how its identity is defined. Often the intent or goals are not necessarily deducible from what people say but from the way they go about doing things.	Value interventions	Changes that transform the underlying logic of business operations and orient activities toward generating human and natural well-being. This reorganizes economic profits from ends to a means that empowers a business to become purposeful endeavors. Businesses may also influence external dynamics by transforming the idea or goal that constitutes a city or a neighborhood. Collectively businesses may reshape the identity of urban areas by establishing hubs for specific activities, shaping the material fabric and mental perception of an area.	**Internal operations** • Reorient internal decision-making towards participatory and equitable procedures; from profit to not-for-profit; focusing on social and environmental objectives as the core of the business model. **External dynamics** • Develop innovation or cultural hubs to alter the perceptions and purposes of neighborhoods.
The mindset or paradigm out of which the system—its goals, structure, rules, delays, parameters—arises					
The power to transcend paradigms					

### Resource interventions tinker with quantifiable characteristics

What we label as internal *resource interventions* have gained increasing attention in research on sustainable entrepreneurship. This has been primarily geared toward measuring, accounting and managing resource consumption and material throughput within the firm (Schaltegger et al. 2012; Maas et al. 2016). For example, this includes interventions that “maximise material productivity and energy efficiency … [doing] more with fewer resources, generating less waste, emissions and pollution” (Bocken et al. 2014, p. 48). Such interventions also turn a firm’s “waste streams into useful and valuable input to other production” and make “better use of under-utilized capacity” to improve efficiency of operation (Bocken et al. 2014, p. 49). Illustrative examples include cleaner manufacturing (Bos-Brouwers 2010; Klewitz et al. 2012), environmental management (Halila 2007; Aragón-Correa et al. 2008) and strategies related to product design and longevity (Bocken et al. 2014) (see also Table 2).

**Table 2:** Illustrative examples of parameter resource interventions

An illustration of an **internal resource intervention** is found in a logistics services firm in Toronto, Canada. As part of upgrading their facility, the entrepreneurs sought cost-savings measures to reduce their energy consumption. The company invested in a set of energy efficient technologies, including more efficient heaters, new air conditioning units, and switching energy provider. This intervention was followed by a set of related measures aimed at improving the environmental profile of the company, including anti-idling measures in transport vehicles, solar-power on the roofs of buildings, and recycling. The firm has since been recognized as a pioneer of low-carbon actions within its industry. An illustration of an **external resource intervention** is provided by a small waste management firm in Rotterdam, the Netherlands. The enterprise has innovated technologies to reduce plastic pollution in open waters. To address this problem, the company developed litter traps to collect plastic waste in rivers preventing it from entering the ocean. The collected plastic is used to manufacture building material for floating hexagonal pods that can be used to provide habitats for native fauna and flora. The company has attracted global attention, which supported it to replicate this intervention in other geographical and cultural settings to reduce plastic pollution and increase water quality and habitats.	

External *resource interventions* refer to entrepreneurship aimed at modifying processes, such as increasing or decreasing the frequency of usage and quantity of physical materials in an urban system. For example, this includes innovations in the context of industrial ecology and its sub-fields, such as industrial symbiosis (e.g., Chertow 2008). It focuses on entrepreneurial actions that create linkages among clusters of firms to repurpose waste from one manufacturing process as valuable resource for another (Staber 1997; Cohen 2006; Desrochers and Sautet 2008). Closed-loop business models, cradle-to-cradle businesses, and life-cycle analyses are based on a similar premise: that reusing and upcycling of materials throughout entire industries and supply chains will guide societies along more sustainable trajectories (Ferguson & Sousa 2010). Related interventions may also change the type of materials that are being used, such as actions to substitute finite resources with renewable energy and “to reduce environmental impact [through creating] significantly more environmentally benign industrial processes” (Bocken et al. 2014, p. 50). The influence of businesses on urban dynamics becomes particularly visual through concepts like urban metabolism, illustrating the ability of firms to tinker with energy and material flows of the city (Lyons et al. 2018; Fróes and Lasthein 2020) (see also Table 2).

### Transactive interventions modify practices

Internal *transactive interventions* target changes in the interactions of people connected with the business, how they engage with its goods and services, and the knowledge of staff members and customers. This focus has grown in research on sustainable entrepreneurship with the recognition that improving efficiency is “necessary, but insufficient to achieve sustainability,” which ultimately requires new practices to “link sustainable production and consumption” to address social equity (Hartman et al. 1999, p. 258). Interventions can range from changing employee practices (Baillette and Barlette 2018) and manufacturing processes (Foerstl et al. 2010) to building new relationships with customers through entrepreneurial innovations that focus on product functionality instead of ownership (Tukker 2004), reducing overall consumption through new incentive schemes (Bolton and Hannon 2016), and integrating production and consumption through prosumer entrepreneurship (Boyaval and Herbert 2018). Similarly, the education of customers and training of employees may be necessary for completing transactive interventions; this creates new avenues for communicating more than just price and function to customers or wages to staff members by signaling who worked on a product or service and in what conditions (Wempe 2005) (see also Table 3).

**Table 3:** Illustrative examples of transactive interventions

An illustration of an **internal transactive intervention** is provided by a sustainability-oriented construction company in Toronto, Canada. The intervention consists of measuring employee waste output and impacts during home renovation jobs. This strategy is fundamental to the personal beliefs of the president, who founded the company with the intention of developing a holistic approach to home renovations that would reduce environmental impact. The business model was extended to the realm of employee practices. Employees are asked to track their work travel, food waste, and types of containers used at work. Employees are encouraged to find alternatives to fossil fuel-based transport and decrease food packaging and waste for lunches and snacks. An illustration of **external transactive intervention** is provided by a social entrepreneur in a property management and design firm in Brixton (London), the UK. The company provides a temporary space for local independent small businesses and community interest companies to learn about entrepreneurship and grow their businesses. This is important because a combination of financial, social, experiential and cultural factors limit these companies from seeking out traditional avenues to start businesses. To support local entrepreneurs in overcoming barriers to development, the property and design firm provides short-term leases at reduced rental rates as compared to market rates, services to assist in developing business plans, and network opportunities to grow, enhance business literacy, and build local community support. One major outcome is that the council, in consultation with the property and design firm, is discussing potential areas to expand this model across the borough.	

External *transactive interventions* focus on changing ‘the way people do things’ in a city to support residents in taking up sustainability-oriented routines through building capacity and supporting cross-sectoral partnerships (Muñoz and Cohen 2016). Traditionally, scholarship conceptualized entrepreneurship as creative destruction (Schumpeter 1934), limiting the transformational role of businesses to disrupt and create new markets (Estrin et al. 2020). Research beyond this narrow understanding of the influence of entrepreneurs on cities is diverse, while some studies have started to explore the various ways through which entrepreneurship influences routines, knowledge, and multi-sectoral collaborations in urban systems (Loorbach and Wijsman 2013; Cohen and Muñoz 2015; Burch et al. 2016). For example, in collaboration with public and academic actors, entrepreneurs can influence daily routines of business delivery and help reschedule related services to off-peak hours to reduce noise and air pollution, increase residents’ quality of life, and lower delivery related costs (Holguín-Veras et al. 2018). Sustainable entrepreneurs can also support the ecological integrity of cities by designing green roofs or other new construction and design approaches; such approaches can be used as strategic tools to foster rethinking what urban environments are used for and support changes in building practices (Loorbach et al. 2009; Schäffler and Swilling 2013). Here, businesses become crucial actors in establishing social networks to advocate for new practices and technologies (Schot and Geels 2008) (see also Table 3). Finally, entrepreneurs also play a crucial role in transforming mass markets (Schaltegger et al. 2016) and the underpinning social processes (Westman et al. 2022).

### Organizational interventions reconfigure agency and power

Internal *organizational interventions* include changes in informal rules that determine the agency and governance of business practices, as well as formal policy and company regulations (Audretsch et al. 2009; Arroyo 2012). This speaks directly to the power that entrepreneurs have on influencing the course of actions within a business. Related phenomena have been addressed in the context of interventions that affect people’s ability to exercise agency as well as through research on collaborative decision-making models and ownership structures (Cheney et al. 2014; Dutt et al. 2016). For example, Stubbs & Cocklin’s (2008, p. 114) work identified a number of attributes under internal organizational capabilities, including revenue sharing, community shareholder ownership and cooperation. Similarly, advanced by organizations such as B-Lab, shared ownership and collaborative decision-making are also taking on increasing importance as indicators of social impact of sustainable entrepreneurs (Rawhouser et al. 2019) (see also Table 4).

**Table 4:** Illustrative examples of organizational interventions

An illustration of **internal organizational interventions** is provided by a small design firm in Rotterdam, the Netherlands. The ‘plastic design company’ is a firm that works to create innovative solutions for plastic waste. Founded in 2013, the firm perceived insufficient communication between themselves and their clients, leading to project delays and other issues. This led to the development of their co-design strategy. The ‘co-design’ strategy is meant to promote close collaboration throughout the entirety of the project, incorporating the client into all stages of the design and building process and creating a collaborative creative process. By conducting projects with a deeper understanding of the goals, values and context of their clients, the company is able to reach mutually satisfactory results that more effectively link the social and environmental values of the client organization to larger environmental issues. An illustration of **external organizational interventions** is provided by a cleaning company in Vancouver, Canada. As a social enterprise, the company was founded with the purpose of providing work opportunities to socially marginalized groups. Being located in an urban area afflicted by socio-economic issues, the enterprise seeks to empower individuals through training and employment. The entrepreneurs also became involved in policy-making processes related to social hiring practices in the city of Vancouver. As a result, the firm is able to help alter formal regulations that determine access to livelihood in the area and diffuse social hiring practices.	

External *organizational interventions* refer to measures that are rarely discussed in previous research on entrepreneurship in sustainability transitions. These interventions change the power of businesses in urban governance and the involvement of firms in making rules and building systems of authority in a city, as well as the ability of businesses to alter patterns of recognition, legitimacy, and accountability of decision-making processes (Pacheco et al. 2010a; Cohen and Muñoz 2016; Oliveira and Hersperger 2018). For instance, private-public partnerships exemplify this reorientation of urban governance, rulemaking, and system building, as firms become service and resource providers for cities (Austin and McCaffrey 2002; Nijkamp et al. 2002; Muñoz and Cohen 2018). In research on sustainability-related decision-making processes, businesses are often depicted as actors exerting a negative influence over socio-environmental dynamics. Large corporations and incumbent industries may hinder the progress of environmental governance (Levy and Newell 2005; Geels 2014), for example, and SMEs may display limited interest in sustainability-oriented policy-making processes (Setzer and Biderman 2013). External organizational interventions, however, draw together disparate knowledge on entrepreneurial innovations and contributions to novel distributions of agency, such as efforts to provide resources and recognition for community interests (Jenkins 2006; Lawrence et al. 2006; Stubbs and Cocklin 2008). Business coalitions at the neighborhood level, such as business improvement areas/districts, aim to better embed firms in the fabric of local communities, contributing to the development of community identity and provisions (Gomez et al. 2015; Zimmermann et al. 2017). This draws attention to the underexplored efforts of entrepreneurs to create recognition and economic participation for socially marginalized and equity-seeking groups or to redefine sustainability-oriented policy discourses (Muñoz and Cohen 2016) (see also Table 4).

### Value interventions change the underlying logics

Internal *value interventions* change the intention or goal of a venture and the values that inform how entrepreneurs operate a business and what activities they pursue (Thompson 2009; Runyan and Covin 2019). Related interventions affect the value creation, proposition, and capture of a business and therefore present a strategic tool to understand and fundamentally alter its sustainability performance (Stubbs and Cocklin 2008; George and Bock 2011; Lüdeke-Freund 2020). The traditional view of entrepreneurs is that their sole intent is to generate profits (Friedman 1962). This is framed as “a necessary condition to stay in the game,” and the ultimate goal of conventional entrepreneurship is said, “to increase market share, to bring the world more and more under the control of the corporation” (Meadows 1999, pp. 16–17). Accordingly, value interventions for sustainability aim to ‘repurpose’ the business, changing the underlying logic in ways so that the firm will by default create social and environmental well-being, as well as refocus the area of activities to the local context instead of creating ever-expanding corporations (Shepherd and Patzelt 2011; Gomez et al. 2015; Lozano 2018). The growth and evolution of these different values is not a process that happens in isolation within a firm, but rather is shaped by broader patterns of political discourse, civic advocacy, and social movements (Castán Broto et al. 2020; Westman et al. 2022). This line of research is still in its infancy. Increasingly scholars explore alternative business approaches, ranging from broad explorations of how organizations can encourage innovations for sustainability (Lüdeke-Freund 2020), to examining particular models such as benefit corporations (Stubbs 2017) or community-based enterprises (Hertel et al. 2019; Luederitz et al. 2021) that repurpose firms as vehicles to support the common good (see also Table 5).

**Table 5:** Illustrative examples of value interventions

An illustration of an **internal value intervention** is provided by a recruiting business based in Toronto, Canada. Since its establishment in the 1950s, the firm has operated as a regular business. However, through a change of leadership in the last decade, the company has fundamentally reoriented its purpose towards providing work with ‘meaning.’ The company radically restructured its internal decision-making processes to empower employees and embed value-driven principles in all business operations, including providing meaningful work, supporting community engagement and volunteering, and shifting to environmental-friendly practices. In 2011, the business became one of Canada’s founding Benefit Corporations (B-corps), and the firm continues to strive to increase its rating in the rigorous B-Corp assessment. An illustration of an **external value intervention** is provided by a group of entrepreneurs in Rotterdam, the Netherlands. The group required an affordable location to sell artisanal food products. After a prolonged search, they obtained permission from the municipality to temporarily occupy and renovate an empty warehouse located in one of Rotterdam’s peninsulas. The collective efforts to establish the ‘entrepreneur market’ quickly extended beyond their warehouse space, leading to changes in administrative procedures and drawing interest from developers. As a collaboratively governed makers-space in a repurposed warehouse, the ‘entrepreneur market’ established a precedent for other initiatives to repurpose vacant spaces. Moreover, it helped significantly to change the perception of the peninsula from a problem area to an area of potential and under-utilized space, resulting in a new collective identity of the neighborhood.	

External *value interventions* change the influence entrepreneurs exert on how a city or a neighborhood is perceived and the identity that defines it (Parzer and Huber 2015; Martucci 2019). The underlying ideas and principles that inform urban development have changed throughout the centuries, but also more recently as visions about radiant cities, garden cities, automotive cities, compact cities, and eco-cities went in and out of fashion (Jabareen 2006; Sharifi et al. 2016). However, the core idea, “that space in downtown cities is enormously valuable,” has rarely changed (Meadows 1999, p. 18). Likewise, the notion of urban centers as engines of economic development has proven remarkably durable (Johnson 2008; Harvey 2016). In contrast, the role of entrepreneurs in supporting sustainability transformations of cities and neighborhoods has only recently gained scholarly attention and continues to be under-researched (Whiteman et al. 2011; Cohen and Muñoz 2015; Gomez et al. 2015). Some studies have eluded to ways through which businesses change the core dynamics of neighborhoods (Hoyt 2005; Charenko 2015). Related research points to the ability of sustainable entrepreneurs to collectively reshape neighborhoods by establishing, for example, high-tech hubs or developing sustainable communities that impact social inclusion, economic activities, and the material fabric (Gomez et al. 2015; Muñoz and Cohen 2016) (see also Table 5).

### Synthesizing internal and external entrepreneurial interventions

Drawing on systems theory and different perspectives on entrepreneurial interventions, we systematically explored the different ways through which entrepreneurship can support sustainability transformations of cities. Based on the scholarship on sustainable entrepreneurship and research on the role of entrepreneurs in urban transformations, we conceptualized two levels of interventions: 1) the level at which sustainable entrepreneurship takes place within a firm (internal) and 2) the level at which entrepreneurship interacts with urban dynamics (external). Building on the concept of leverage points, which is based on observations that small interventions in one area can lead to transformations in multiple system characteristics, we theorized entrepreneurial interventions on both levels. We proposed four interventions on each level to categorize change processes and offer a more fulsome conceptualization of the transformational potential of how entrepreneurship can contribute to urban transformations. The four interventions include: 1) resource interventions that target ‘mechanical’ system properties, changing quantifiable indicators and physical structures; 2) transactive interventions that target how the system functions, changing interactions and practices; 3) organizational interventions that target the governance of the system, modifying the agency people have in change; and 4) value interventions that target the underlying goals and mindset of systems, changing the identity and values that shape the nature of the other four intervention points (see Table 6). Enriched with empirical illustrations, we showed the practical application of the proposed framework, demonstrating the various ways through which entrepreneurs drive sustainability change internally and externally.

**Table 6:** Interventions through which businesses can support sustainability transformations

	Resource interventions	Transactive interventions	Organizational interventions	Value interventions
**Internal change**	Related interventions adjust material and quantifiable elements of existing rates, size or the nature of resource consumption in a business.	Related interventions change interactions, behavior, and knowledge of people connected with the business, including its relationship with suppliers and customers.	Related interventions reshape the organization of the business by redefining decision-making authorities of customers, employees, and owners, and modifying the agency people have in change.	Related interventions change the core purpose and goal of a business (i.e., why a company does business) and the values that inform how a business is operated and what it does.
**Illustration of internal change**	• Reduce, recycle, reuse resources • Increase energy efficiency • Change to renewable resources	• New customer reward systems • Employee training • New employee behavior, for example, car-sharing	• Establishing new positions in the company • Providing new resources or decision-making powers to employees • Redefining roles of clients in company decisions	• A company shifts from a for-profit to a not-for-profit business model • A company begins to place social or environmental objectives at the core of the business model
**External change**	Related interventions change the size or rate of existing processes or material structures in a city, such as modifying the production, consumption or flows of physical materials in the city	Related interventions changes ‘the way people do things’ and interact in a city, including daily habits and routines, as well as knowledge and relations between actors	Related interventions change who gets to decide on the rules and authority in a city, and alter the legitimacy and accountability of decision-making processes.	Related interventions change what constitutes core ideas and “goals” of a city, such as the identity of a neighborhood, who it serves, or the beliefs about a city
**Illustration of external change**	• Reduce city-wide waste • Increasing water quality and natural habitats	• A business changes practices in the construction industry by providing sustainable building solutions • A business changes the food production in a city by disseminating vertical farming practices	• Influences changes to regulatory standards that improve access to livelihood in a community • Introduces a new advisory board that gives businesses leverage over public policy • Involves a firm in decisions about or the management of public resources	• Firms contribute to establishing a neighborhood as a cultural hub of the city • Firms contribute to the image of sustainable neighborhoods

In Table 6, we summarize the four interventions through which the transformational potential of entrepreneurship is realized and specify how they contribute to changing a firm’s own operations (internal influence) and the urban system (external influence). The interventions are arranged based on the changes they generate, including resource interventions, transactive interventions, organizational interventions, and value interventions. Underling this arrangement is the observation that, as one moves in Table 6 from left to right, interventions increasingly influence changes in previous categories (see also Fig. 1). For example, transactive interventions that change practices (e.g., carpooling of employees) also affect resource interventions, as fewer vehicles are used and fewer emissions are generated. Similarly, value interventions that support businesses to generate ecological and social well-being often require changes in organizational interventions (e.g., changing decision-making structure to empower employees) and transactive interventions (e.g., changing routines and manufacturing practices) to reduce environmental and social harm (resource interventions).

## Discussion

Our conceptual framework (Fig. 1 and Table 6) organizes entrepreneurial interventions and assesses their potential to support urban sustainability transformations. We advance this analysis by proposing *pathways of change* to theorize how business interventions are connected and co-evolve to generate sustainability transformations. Taking the business organization as the focal point, we first examine ‘horizontal’ interconnections of entrepreneurial interventions. By drawing on insights from research on sustainable entrepreneurship, we conceptualize how a given entrepreneurial intervention may trigger a pathway of change across intervention types within firms. Second, we examine how pathways of change unfold ‘vertically,’ involving changes in the business and on the city-level, by drawing on the principle of co-evolution from sustainability transition scholarship. Together these considerations offer theoretical vantage points to explain why and how entrepreneurship-driven change can travel across system levels and advance transformations.

### From isolated to comprehensive change: ripple effects across entrepreneurial interventions

The transformational potential of entrepreneurship is contingent on its ability to move from isolated interventions in one area of a business toward comprehensively changing the entire organization. Most research compartmentalizes firms’ internal processes or limits the analysis to interactions between firms, but rarely investigates how isolated activities connect across intervention types and collectively change the sustainability performance of a business. For example, entrepreneurial activities that support ‘pro-environmental behavior’ of employees (e.g., reducing carbon emissions through changed practices) can trigger change beyond the immediate boundaries of the firm (e.g., in the supply chain) (Shrivastava 1995; Kennedy et al. 2015). The proposed framework allows detailed examination of how an isolated intervention is connected to broader change. To illustrate, we return to the logistics firm presented in Table 3, where investments into new equipment reduced energy consumption (an internal resource intervention). Over time, as the firm recognized the benefit of this investment, more extensive measures were considered to save resources, which involved behavioral change of employees (internal transactive interventions). Similarly, receiving external recognition as a low-carbon leader following these interventions supported changes in decision-making arrangements, integrating environmental considerations in the strategic orientation of the firm (an internal organizational intervention).

Interventions that target the organizational or value structure of a business frequently trigger change in other intervention types such as resource and transactive structures (this is why they are placed higher up on the seesaw in Fig. 1). For example, alternative business models can change how an entrepreneur goes about doing business and eventually result in substantially reduced resource consumption (Wells 2017; Stubbs 2019; Lüdeke-Freund 2020). This pathway of change from value to resource interventions is illustrated by the construction firm discussed in Table 3. In this case, our empirical research revealed how changes resulted from underlying principles that guided the founding of this business (conducting home renovation with minimal environmental impact). Here, the beliefs and values (internal value intervention) that informed entrepreneurial action led to interventions that changed the day-to-day routines of employees and supported their adoption of environmental-friendly practices (internal transactive interventions).

Research on pathways of change can help trace and structure the process through which interventions within business organizations realize the transformational potential of sustainable entrepreneurship. We posit that if pursued in isolation, the four intervention types contribute little to shifting business operations toward sustainability. A systems perspective suggests the need for change across the entire spectrum that the four intervention types target. Put differently, entrepreneurial interventions only become transformational if, aside from modifying the targeted characteristic, they also generate change in other areas of the firm. Understanding the transformational potential of entrepreneurship, therefore shifts research to the innerworkings of businesses to examine the organizational processes and practices within firms. Indeed, social practices constitute the building block of how businesses function as an entity. Greater appreciation of the social dimension is needed to reveal the agency involved in transforming business organizations and their strategic orientation toward sustainability (Luederitz et al. 2021; Westman et al. 2019).

### Urban sustainability: co-evolutionary dynamics of entrepreneurship–city relationships

A key dynamic of urban transformations that the proposed framework allows us to conceptualize is the interaction between entrepreneurial interventions and the urban context. The framework conceives of businesses and urban systems as nested levels. Accordingly, insights from co-evolutionary perspectives suggest that interventions on one level may influence and trigger shifts on the other level (Shrivastava 1995; Starik and Rands 1995; Pacheco et al. 2010b, 2014; Cohen and Muñoz 2015). The framework supports the empirical examination of this phenomenon to systematically explore how interventions that change the business context simultaneously affect the city-level.

To better understand the bidirectional influence between businesses and the city, we suggest drawing on sustainability transition scholarship to help reveal and trace the multiprong role of entrepreneurship. Past research suggests that for entrepreneurship-driven innovations to emerge, supporting social processes associated with the adoption of new practices or technology are required, including the establishment of material structures, markets and industries, policies and regulations, and user practices (Geels 2005; Johnson and Schaltegger 2019; Westman et al. 2022). Mechanisms through which co-evolution is realized include network learning, collective norm-construction, and collaborative advocacy (Westman et al. 2022). These dynamics support the diffusion of practices and learning across multiple actors, allowing interventions to spread beyond the initial protective space.

The proposed framework can mobilize this co-evolutionary understanding to capture and examine business-city relationships and how interventions co-evolve in urban transformations. For example, our empirical research examined a Toronto-based food retail entrepreneur that has been able to influence external dynamics through network learning, collective norm-construction, and collaborative advocacy. First, the food entrepreneur collaborated with local farms and producers (by sourcing, provision of funding, and training) to implement organic farming and other practices with low environmental impact in a number of firms (a transactive intervention). This resulted in expanding the group of companies that apply environmentally sustainable production methods in Toronto. Second, the entrepreneurs worked with local community groups and other civil society organizations to educate the public on various aspects of sustainable food production and consumption (a transactive intervention). Third, the entrepreneurs established a policy advocacy group that has contributed to the adoption of sustainable food standards (an organizational intervention). Through these activities, the firm has contributed to the construction of pathways of change to support changes on the city-level and build a sustainable food system through shifts in practices throughout supply chains, altered customer behaviors, and institutional frameworks.

Future research and empirical investigations into how entrepreneurship shapes urban transformations are warranted to critically examine such co-evolutionary dynamics. The proposed framework supports such analysis as it conceptualizes the influence that businesses can exert on their city environment. Linking the proposed framework with sustainability transition scholarship for a systematic examination of pathways of change would support research on how individual business interventions contribute to transformations of urban environments. This perspective would contribute to better understanding why certain entrepreneurial interventions fall short of realizing the transformational potential built into their endeavors. As an ex-ante tool, the framework could also aid reflection on how to design and intervene in complex urban systems, ensuring entrepreneurial success while challenging characteristics that maintain unsustainable dynamics and navigating social arrangements unfriendly to sustainability innovations. While our analysis was limited to urban environments with case studies drawn from cities in Europe and North America, enriching and complementing the framework would require research beyond this context.

For developing and specifying the presented framework, we chose as our starting point entrepreneurs and explored ripple effects within businesses and their bidirectional relationship with cities accordingly. Given the nested nature of these two levels, urban transformation scholarship can also rely on the presented framework to explore how city-level interactions frame, demarcate, and support entrepreneurial interventions within businesses. For example, in our research we identified various arenas in which public administrations had provided spaces for entrepreneurs to work on urban sustainability innovation. Similarly, ripple effects within the city-level could be explored through our framework. For example, our empirical data comprised observations of neighborhoods and city districts that over time changed the underlying logic of functioning by redefining its core identity. Analyses of how such changes ripple through, affect, and co-evolve with other intervention types could also be aided through the presented framework.

## Conclusion

The significance of the developed framework lies in moving the analysis of entrepreneurship beyond detailing the innumerable initiatives that shape urban transformations and conceptualizing the multi-pronged role of entrepreneurs in processes of system-wide change for sustainability. Thus, the framework is not a mechanistic toolbox but rather an invitation to critically reflect on the transformational potential of entrepreneurship for sustainability transitions and how to effectively support its realization. Ultimately, the transformational potential of entrepreneurship is contingent on its ability to change the properties that are targeted, and initiate ripple effects throughout a given system. Structuring interventions through our framework offers a systematic way to grapple with the perplexing reality of a multitude of ongoing sustainable business initiatives that seemingly fail to generate transformational change. Our framework helps to theorize this phenomenon by analyzing the specific nature of resulting change. For example, our literature review revealed that most research focuses on resource and transactive interventions with far less attention being devoted to change that questions and redefines how businesses are organized or why businesses do business (organizational and value interventions). As a result, business interventions that target resource and transactive interventions (often directed to material and technological change only) may struggle to create fundamental system reconfigurations, despite being heralded as radical innovation.

The presented framework supports future research in critically examining the nature of entrepreneurship, how interventions are performed and the resulting change that is achieved. We call attention to the importance of examining *pathways of change* that describe how entrepreneurial interventions are connected with and co-evolve across nested levels of action to generate sustainability transformations. Aided by the framework, future research could examine pathways of change that result and are accelerated by entrepreneurship to systematically appraise if and how interventions realize their transformational potential and identify conditions that support such efforts. This will allow tracing how transformations unfold as entrepreneurs build on initial success in one area to leverage change in other areas of the organization and at the city-level.

Ultimately, this framework can serve as a heuristic tool for researchers to critically reflect on the transformational potential of the flurry of entrepreneurial interventions currently underway. A critical examination of the multitude of ways that support the realization of the transformational potential of entrepreneurship can function as a strategic compass for practitioners and researchers to both rigorously examine and explicitly support change for urban sustainability.

## Data Availability

The raw data is not available due to confidentiality agreements.
